# Providing immediate neonatal care and resuscitation at birth beside the mother: parents’ views, a qualitative study

**DOI:** 10.1136/bmjopen-2015-008495

**Published:** 2015-09-16

**Authors:** Alexandra Sawyer, Susan Ayers, Sophia Bertullies, Margaret Thomas, Andrew D Weeks, Charles W Yoxall, Lelia Duley

**Affiliations:** 1 Centre for Health Research, School of Health Sciences, University of Brighton, Brighton, UK; 2 Centre for Maternal and Child Health Research, City University London, London, UK; 3 Neonatal Unit, Liverpool Women's Hospital, Liverpool, UK; 4 Department of Women's and Children's Health, University of Liverpool, Liverpool, UK; 5 Nottingham Clinical Trials Unit, University of Nottingham, Nottingham, UK

**Keywords:** NEONATOLOGY, QUALITATIVE RESEARCH

## Abstract

**Objectives:**

The aims of this study were to assess parents’ views of immediate neonatal care and resuscitation at birth being provided beside the mother, and their experiences of a mobile trolley designed to facilitate this bedside care.

**Design:**

Qualitative study with semistructured interviews. Results were analysed using thematic analysis.

**Setting:**

Large UK maternity hospital.

**Participants:**

Mothers whose baby received initial neonatal care in the first few minutes of life at the bedside, and their birth partners, were eligible. 30 participants were interviewed (19 mothers, 10 partners and 1 grandmother). 5 babies required advanced neonatal resuscitation.

**Results:**

5 themes were identified: (1) Reassurance, which included ‘Baby is OK’, ‘Having baby close’, ‘Confidence in care’, ‘Knowing what's going on’ and ‘Dad as informant’; (2) Involvement of the family, which included ‘Opportunity for contact’, ‘Family involvement’ and ‘Normality’; (3) Staff communication, which included ‘Communication’ and ‘Experience’; (4) Reservations, which included ‘Reservations about witnessing resuscitation’, ‘Negative emotions’ and ‘Worries about the impact on staff’ and (5) Experiences of the trolley, which included ‘Practical issues’ and ‘Comparisons with standard resuscitation equipment’.

**Conclusions:**

Families were positive about neonatal care being provided at the bedside, and felt it gave reassurance about their baby's health and care. They also reported feeling involved as a family. Some parents reported experiencing negative emotions as a result of witnessing resuscitation of their baby. Parents were positive about the trolley.

**Strengths and limitations of this study**

- This is the first study to explore parents’ experiences of neonatal care and resuscitation being provided beside the mother. Use of in-depth qualitative methods allowed parents’ experiences to be explored in detail.
- Limitations include that participants were primarily white, married or living with their partner, the babies were all alive at the time of interview, parents were recruited from a single site, which had pioneered this type of care, most of the babies did not need advanced resuscitation at birth, and the interviews were largely conducted in hospital before the baby had been discharged home.
- It is possible that parents may have been reluctant to criticise their care or use of the trolley. However, none of the interviewers were involved in their care at birth or their baby's care.

## Introduction

In the UK, approximately one-third of births are attended by someone trained in newborn life support. For most babies, the care they receive during this immediate period is an assessment of their condition, to determine whether or not resuscitation is needed, and drying and wrapping to prevent hypothermia. A minority of babies will also require some degree of resuscitation, ranging from simple airway opening manoeuvres to full cardiopulmonary support.^1^ Usual practice is that the baby is taken to a resuscitation platform at the side of the room, or in another room, for this assessment and initial neonatal care. While the baby is being cared for on the resuscitation platform, the mother and her partner are not able to see their baby or know what is happening.^2–4^

Family presence during resuscitation of adults and children is often preferred by families and can be beneficial.^5–10^ One study found that the majority of parents believed that being there and being able to touch their child provided comfort to their child, and helped the parents to adjust in the event of the loss of the child.^11^ Only one study has explored parents’ experiences with newborn resuscitation and this was with fathers.^12^ Fathers’ recollections of resuscitation were mainly negative, including feelings of worry, distress and fear; however, they did not express regret at being present. Central to fathers’ experiences was a conflict over whether to stay with their partner or go to see their baby on the resuscitation platform. This highlights the unique issues associated with family presence during neonatal resuscitation, the importance of exploring parents’ experiences, and that bringing neonatal care to the mother's bedside may help parents deal with this difficult situation.

As part of a programme of work to improve outcome and quality of care following preterm birth, we developed strategies for providing newborn life support at birth beside the mother. The aims were to allow parents to share the first moments of their baby's life and, as part of planning a randomised trial of deferring cord clamping for very preterm births, to assess whether newborn life support with the umbilical cord intact is possible.^13^ We showed that providing neonatal care beside the mother can be carried out successfully either with a mobile trolley designed for this purpose (BASICS; Bedside Assessment, Stabilisation and Initial Cardiorespiratory Support; marketed as LifeStart),^14^ ^15^ or by moving and adjusting the standard resuscitation equipment.^16^ A previous study with clinicians who have provided neonatal care beside the mothers using the mobile trolley found that clinicians felt the trolley improved parents’ overall experiences.^14^ To assess parents’ own views and experiences of care at the bedside using the mobile trolley, and to determine whether witnessing resuscitations had any negative impact on parents, we conducted this qualitative study. Views and experiences of clinicians are reported in a separate paper (CW Yoxall, S Ayers, A Sawyer, *et al*. Providing immediate neonatal care and resuscitation at birth beside the woman: clinicians’ views, a qualitative study. *BMJ Open*, submitted).

## Methods

Recruitment took place between November 2012 and January 2014 in a maternity hospital, where the trolley was developed and where it was introduced into clinical service in November 2012. The trolley then underwent a period of service evaluation, which confirmed that it is possible to provide all of the newborn resuscitation interventions beside the mother, and that this appeared to be broadly acceptable to clinicians and the mothers.^14^ For this qualitative study, purposive sampling was used, where all eligible parents whose babies had initial care in the first few minutes of life at the bedside using the trolley were invited to take part in the study. These were likely to be mothers who would normally expect to have an advanced neonatal nurse practitioner or paediatrician in attendance at birth (eg, all non-elective caesarean sections, all births under 36 weeks gestation). In the UK, only babies for whom there are risk factors (eg, prematurity, congenital abnormality, abnormal fetal heart trace) or for whom there are initial health concerns, would be managed on a resuscitation unit. This study includes parents of babies in the latter group only. All participants were approached in person and provided with written information. Written informed consent was obtained from each participant. Semistructured interviews were carried out by either a female psychologist (PhD) or midwife trained in qualitative methods, and each interview lasted approximately 30 min. The interviewer would introduce herself and explain the purpose of the research. All participants spoke English well. At the time of the interviews, 17 mothers’ babies were in hospital and two had been discharged. None of the interviewers were involved in the babies’ care or their care at birth. Interviews took place either in a private room in the postnatal ward or at the parent's home. No one else was present apart from the interviewer. Interviews were recorded and then transcribed with all identifying information removed. Data collection ended when data saturation had been achieved.

The interview schedule consisted of open-ended questions, which were used as a guide to explore parents’ experiences with the trolley, their experience of neonatal care at birth, and if their baby had not required resuscitation at birth how they might feel about being so close if their baby had been very seriously sick at birth. The interviewer had the freedom to probe the interviewee to elaborate on responses or follow a line of inquiry introduced by the interviewee. Cues and prompts were also used by the researcher to allow the interviewee to discuss the topic further. Basic demographic information was extracted from the maternal and neonatal case notes.

### Data analysis

Qualitative analysis of the transcripts used inductive thematic analysis described by Braun and Clarke^17^ to identify, describe and analyse themes and patterns within the data. First, transcripts were read and reread to familiarise the researchers (AS and SB) with the data. Second, all interviews were coded in detail to ensure all codes arising were included in an initial pool of codes. Third, the pool of codes was sorted into potential themes on the basis of frequency, significance and overlap. Where there were overlaps between codes, these were collated into themes or subthemes. Fourth, themes were reviewed by authors (AS, SA and SB) in relation to the generated codes and the entire data set. Finally, themes were named and defined in a coding schedule, which was used to code all interviews again to ensure consistency of coding. NVivo V.10 software (QSR International Pty Ltd) was used to organise codes and themes.

For this report, direct quotes are coded (number=participant number; Mother/Partner; Term=Term Birth, Preterm=Preterm Birth; type of resuscitation) to ensure anonymity.

## Results

Of the 56 mothers and their birth partners approached, 30 were interviewed (19 mothers, 10 partners, and 1 grandmother). Although 30 participants were interviewed, the total number of interviews conducted was 19 because all the mothers chose to be interviewed with their birth partner. The mothers were mostly white European, and were either married or cohabiting (see table 1). Their age ranged from 19 to 39 years (*median*=28). Five babies required advanced neonatal resuscitation at birth.

**Table 1:** Demographic information of the mothers and details about the birth

	*N*=19 (%)
Ethnicity	
White European	17 (90)
Asian	1 (5)
African	1 (5)
Marital status*	
Married/living with partner	11 (69)
Single†	5 (31)
Employed	7 (37)
Parity	
Nulliparous	12 (63)
Multiparous	7 (37)
*Birth details*	
Gestation at birth (weeks)	
Term (37+)	7 (37)
Moderately preterm (32–36)	4 (21)
Very preterm (<32)	8 (42)
Type of birth	
Vaginal	11 (58)
Caesarean (elective)	6 (31)
Caesarean (emergency)	2 (11)
Multiple birth	1 (5)
Advanced resuscitation at birth	5 (26)
Baby admitted to NICU	13 (68)
Place of interview	
Hospital	17
Home	2

*Owing to missing data n=16.

†All of these mothers had a birth partner present.

NICU, neonatal intensive care unit.

Five themes were identified in families’ experiences of care at the bedside: (1) Reassurance; (2) Involvement of the family; (3) Staff communication; (4) Reservations and (5) Experiences of using the trolley (see table 2).

**Table 2:** Themes, subthemes and sample quotes from the 19 interviews with parents

Theme/subtheme	Sample quotes
1. Reassurance	
Baby is OK	“and that he was moving, so much reassurance” (*68; Partner; Preterm; Cardiac massage*)
Having baby close	“When he eventually was fully born, they pulled him out and placed him on the trolley and, from a father's point of view, it was a thousand times better than having him further away” (*90; Partner; Term; Airway suction*)
Confidence in care	“being able to see what they were doing and reassurance that he was been taken care of and they were doing the best they could” (*68; Partner; Preterm; Cardiac massage*)
Knowing what's going on	“there would be a big comfort there when you could actually see what was going on properly” (*2; Mother; Term; Dry and cover*)
Dad as informant	“It worked perfectly for us, because he could see and then he could reassure me” (*90; Mother; Term; Airway suction*)
2. Involvement of the family	
Opportunity for contact	“it was really nice that he could be there and I could still see him instead of just taking him away right away” (*2; Mother; Term; Dry and cover*)
Family involvement	“It was more by watching them that I knew what was going on than what I was being told” (*68; Partner; Preterm; Cardiac massage*)
Normality	“So it's nice having her just that bit closer to her and be able to hold her hand and getting a bit of normality out of birth, it's not all traumatising. There's that little tiny bit of goodness to have her there and see” (*88; Mother; Preterm; Surfactant*)
3. Staff communication	
Communication	“and that was all really for the trolley in a way because each bit of it was kind of…I think you felt more because you could see the process, they would explain the process” (*90; Partner; Term; Dry and cover*)
Experience	“The only thing I can remember was the anaesthetist who was standing behind us say ‘what's that there for?’ and ‘whose is that?’ and ‘what they doing?’ and everyone was talking about it in the room as they were lying me down. All the doctors were all talking and explaining to each other what it was and what it was there for” (*13; Mother; Term; Dry and cover*)
4. Reservations	
Reservations about witnessing resuscitation	“It would have scared me, it would have frightened me. I mean, I would have wanted to obviously see so I knew he was alright but at the same time any normal person would be scared” (*3; Mother; Term; Dry and cover*)
Negative emotions	“It is quite stressful seeing doctors working like that” (*68; Partner; Preterm; Cardiac massage*) “It's a stressful scary process, isn't it, watching things happen to your baby like that” (*92; Mother; Preterm; Surfactant*)
Worries about the impact on staff	“Because there is also the side of it that there is a lot of pressure on them that they are, like things turned out particularly well with us, but for some babies who might need more work it's a lot of pressure on them trying to resus a baby in front of mum. I think that must be very, very hard” (*62; Mother; Term; Mask ventilation*)
5. Experiences of the trolley	
Practical issues	“They had to cut the cord because it was too far away physically to get her to [the trolley]” (*62; Partner; Term; Mask ventilation*) “If she was on the little one I wouldn't have seen it anyway because of the big screen” (*66; Partner; Preterm; Cardiac massage*)
Comparison with standard resuscitation equipment	“They looked quite scary—the big resuscitaire ones” (*52; Mother; Preterm; Dry and cover*)

Although 30 participants were interviewed, the total number of interviews conducted was 19 because mothers were interviewed with their birth partners.

Number=participant number; Mother/Partner; Term=Term Birth; Preterm=Preterm Birth; Type of resuscitation.

### Reassurance: “I would have felt worse if they had taken him away and then not knowing”

This theme contains five subthemes and describes how parents who witnessed either lower intensity interventions or resuscitation felt reassured because they could see their baby and the treatment he/she was receiving. The first subtheme, ‘Baby is OK’, refers to parents feeling reassured about their baby's health. Many parents felt reassured by having their newborn near them and seeing that their baby was alive, moving or physically improving (*68; Partner; Preterm; Cardiac massage*).

Because you know he's ok, you can see him coming round if the resuscitation works, then you can see your baby then can't you, surviving it. (94; Mother; Preterm; Dry and cover)

The second subtheme, ‘Having baby close’, refers to parents feeling reassured that their baby was close to them. Parents reported that they liked having their baby close by (*90; Partner; Term; Airway suction*) and some mothers thought that having the baby taken away would have been more distressing:

Had they taken her away—yes I think I would have probably got a bit more distressed because it is like ‘Why are you taking her over there and what's wrong?’ (62; Mother; Term; Dry and cover)

The third subtheme, ‘Confidence in care’, refers to the reassurance parents felt regarding the care of their baby. Seeing the baby's treatment reassured them that staff were doing the best they could (*68; Partner; Preterm; Cardiac massage*).

But because they had been doing it while I was watching I could actually see they were really trying to help him, they really did help him a lot. (83; Mother; Preterm; Intravenous drugs)

The fourth subtheme, ‘Knowing what's going on’, describes the reassurance parents felt by knowing what was going on (*2; Mother; Term; Dry and cover*).

Because you know what they are doing. You can see exactly what they are doing and if he needs anything, you can see exactly what they are putting on. (94; Mother; Preterm; Dry and cover)

At some births, however, the mother was unable to see the baby due to the position of the resuscitation trolley. The final subtheme ‘Dad as informant’, describes situations where fathers were able to relay information about what was happening, and this reassured and comforted the mother (*90; Mother; Term; Mask ventilation*).

I was saying “he's breathing I can see he is breathing OK. You can't hear him because he has got mask on his face”—she still didn't believe me, but if I hadn't been able to say that she would have panicked a lot more than she did. (90; Partner; Term; Mask ventilation)

### Involvement of the family: “You feel a part of it”

This theme contains three subthemes and describes parents who witnessed either lower intensity interventions or resuscitation, feeling involved as a family. The first subtheme, ‘Opportunity for contact’, describes the opportunity parents had for contact with their baby immediately after he/she was born. Providing care at the bedside meant that they had opportunity for contact and were able to see or touch their baby or hold his/her hand (*2; Mother; Term; Dry and cover*). If they were unable to touch the baby while he/she was receiving care, they said that watching was the next best thing:

Yeah it was good because obviously I wouldn't be able to hold him while they work on him and that was as close as he was going to get. (92; Mother; Preterm; Surfactant)

 The second subtheme, ‘Family involvement’, describes the involvement of parents with their baby and their baby's care. For example, it was by watching that some parents said they understood what was happening to their baby and felt part of his/her care (*68; Partner; Preterm; Cardiac massage*). However, in 11 births, parents were unable to see their baby on the trolley, either because there was a screen up for a caesarean section or because of where the trolley was positioned in relation to the mother, who was unable to sit up:

The screen was so big, we didn't actually see the trolley then that you were testing out. (2; Mother; Term; Dry and cover)

In some of these cases, the father was able to see and could relay information to the mother, and the mother reported that this provided some reassurance. However, one father who attended a caesarean birth said that he was unsure whether he was allowed to come closer to the baby. The final subtheme, ‘Normality’, describes that care beside the mother feels more normal. One mother mentioned that having her baby close at birth gave some “normality” and “goodness” to a birth she otherwise experienced as unnatural (*88; Mother; Preterm; Surfactant*).

Definitely, it's just that one thing to make it more natural because it isn't natural that you're going so early. It just makes it that little bit more of a natural to you the fact that she's close if you understand what I mean. (88; Mother; Preterm; Surfactant)

### Impact on staff: “Everything they explain”

This theme contains two subthemes: ‘Communication’ and ‘Experience’. The first subtheme refers to parents’ perceptions of the impact that providing care beside the mother has on staff communication with the family. Some parents felt that watching the initial neonatal care assisted staff's communication with them because they were so close to the parents and the parents could see what was happening (*90; Partner; Term; Dry and cover*). However, some parents said that they would have liked more explanation about what was going on and why, as this might have allayed some of their fears:

I know they are really busy, in many respects they've got a lot of things to do—like keeping a baby alive, but I think from the patient's perspective, if you can call me a patient and I think some sort of helpful explanations of what they are doing and why would probably allay a lot of people's fears. (62; Partner; Term; Dry and cover)

The second subtheme refers to parents noting whether or not the staff appeared experienced or confident at using the trolley. A few parents commented that staff appeared to be inexperienced in using the trolley, as they noticed some staff still had to work out where to position it (*13; Mother; Term; Dry and cover*). However, one parent thought the staff members using the trolley appeared very confident:

Perfect, so professional, so competent, they knew exactly what they were doing. (52; Partner; Preterm; Dry and cover)

### Reservations: “It's a stressful scary process”

This theme describes parents’ worries about witnessing a resuscitation or lower intensity interventions and contains three subthemes. The first subtheme, ‘Reservations about witnessing resuscitation’, explores parents’ varied views regarding witnessing the neonatal care their baby received. For example, parents of babies who needed low-intensity interventions at the bedside, such as drying and receiving oxygen by mask, reported no reservations, or that they were scared but would like to watch again (*3; Mother; Term; Dry and cover*). However, half of these parents thought they *might* have reservations about watching if their baby had needed more intensive interventions:

Because I am sort of thinking now how would I have felt if they had to resuscitate her and she's have been that close, I think that would have been too much for me. (62; Mother; Term; Dry and cover)

In contrast, none of the parents who witnessed more intensive intervention, such as intubation and cardiac massage expressed regrets about watching. The second subtheme, ‘Negative emotions’, describes the negative emotions reported by parents as a result of witnessing resuscitation. These parents reported some negative feelings, such as being scared or finding their baby's intervention unpleasant to watch (*68; Partner; Preterm; Cardiac massage* and *92; Mother; Preterm; Surfactant*), but 3/5 stated that at the same time it was also “fine” or “nice”. Perhaps because of these mixed reactions, a few parents suggested parents should be asked beforehand whether they would like to watch the neonatal care at birth:

I think the best thing you could do is say to them before they go in is ‘Look at the time of the birth most, a lot of babies get resuscitated would you rather us pull a screen down while we do this’ […] or would you rather us do it in front of you? I think you should get asked the question before you go in. (66; Partner; Preterm; Cardiac massage)

The final subtheme, ‘Worries about the impact on staff’, describes parents’ worries about the impact of being so close to the mother on staff. For example, two parents were also concerned their watching might have an impact on the staff in terms of adding pressure or distraction (*62; Mother; Term; Mask ventilation*):

I didn't want to look away or ask questions so they would be distracted from what's going on. (68; Mother; Preterm; Cardiac massage)

### Experiences of the trolley: “I think they are quite scary looking, the big machines compared to a little trolley”

This theme describes parents’ experiences and opinions specifically related to the trolley. Of the 11 parents who gave an overall opinion of the trolley, 9 commented favourably. Two parents were not sure: one mother stated that if it was “good for baby it's fine”, and one father had reservations about the trolley's usefulness.

The first subtheme, ‘Practical issues’, describes observations made by some parents regarding circumstances in which they thought the trolley might not be useful. For example, two parents mentioned that the cord had to be cut because it was too short to reach the trolley (*62; Partner; Term; Mask ventilation*). Another mother mentioned she would not have been able to see her baby on the trolley because of the screen:

If she was on the little one I wouldn't have seen it anyway because of the big screen. (66; Partner; Preterm; Cardiac massage)

The second subtheme, ‘Comparison with standard resuscitation equipment’, describes how parents perceived the trolley in comparison to the standard resuscitation equipment. The standard resuscitation equipment was also in the room and some parents thought this equipment looked ‘scary’, clinical, or as if their baby needed a lot of help or would be taken away (*52; Mother; Preterm; Dry and cover*). However, two parents felt that the standard equipment looked more advanced:

The initial impression is that it looks quite basic, because you see the big one with the light and everything over it and it's always the one you see on TV so to suddenly see this kind of little trolley… (68; Mother; Preterm; Cardiac massage)

## Discussion

The aim of this study was to explore parents’ views and experiences of immediate neonatal care and resuscitation at birth beside the mother, and of the trolley used to provide this. Providing care and resuscitation at the bedside offered the opportunity for parental contact and involvement with the baby, so they could share the first moments of their baby's life. This was valued by parents. A similar theme was reported in interviews with clinicians (CW Yoxall, *et al*. submitted). Most clinicians commented that providing immediate care at the bedside allowed the parents to witness and even interact in the first moments of their child's life. Usual practice for babies needing assessment and immediate neonatal care is either to take the baby to the side of the room or to another room nearby. So parents often do not see or touch their baby at birth, which may contribute to stress and worry.^2–4^ Touch is important for the parent–baby bond. Parents who have given birth very preterm report immediate bonding when they first touch their babies, although this first touch was in the neonatal unit.^2^ The study presented here shows that immediate care at the bedside also allowed fathers to feel more involved in the birth. Previously, fathers have reported feeling a conflict between whether to stay with the mother or go with the baby to the standard resuscitation equipment away from the mother.^12^ Clearly, providing care at the bedside allows the father to see the baby but also to stay with the mother. For some mothers, being close to their baby may also mean the birth feels more normal. Previous studies with mothers of low birthweight babies have shown the mothers report feeling guilt over the loss of natural contact during birth.^18^ ^19^ The close proximity of the baby reassured parents because they could see their baby and the treatment being given. This is similar to parental presence during paediatric procedures, as parents report that that being present helped them understand resuscitation and see that everything was being done to save their child.^20^

Although parents were generally positive about bedside care, some reported experiencing negative emotions as a result of witnessing the resuscitation. However, no parents reported regret at witnessing their baby being stabilised; which is consistent with a previous study of fathers’ experiences of newborn resuscitation.^12^ Some parents were concerned about the impact on staff of having to perform resuscitation so close to the parents. They thought that staff might perceive parents as evaluating their performance, and were concerned that staff could be distracted by worrying about the parents. A similar concern was raised by clinicians for less-experienced staff (CW Yoxall, *et al.* submitted).

This study has a number of potential clinical implications. Although there are guidelines and recommendations for supporting families during child and adult resuscitation,^21^ ^22^ there is little guidance on support for neonatal resuscitation at birth. In our study, explanation of what was happening during procedures was important to some families. If parents are with their baby during immediate neonatal care or resuscitation at birth, staff should explain to them what they are doing and why.^12^ Consistent with studies of family presence during resuscitation in other populations, it is suggested that parents are asked if they want to witness neonatal care at birth.^9^ ^23^ Current guidelines for neonatal resuscitation state that parents should be encouraged to touch their baby soon after resuscitation.^24^ Provision of initial care at the bedside enables parents to touch their baby during resuscitation. This means parents are involved in the first moments of their baby's life, which is consistent with the principles of family-centred care. A recent randomised controlled trial found that symptoms of distress were higher up to 1 year after the event in family members who did not witness a resuscitation, compared with those who did witness resuscitation.^25^ ^26^ Although this was adult resuscitation, it is possible that witnessing resuscitation of their baby may also reduce feelings of distress for parents. Therefore it is important that methods of enabling bedside care are developed and evaluated. Although bedside care in this study was provided with the trolley, it is also possible to provide bedside care by moving and adjusting the standard resuscitation equipment.^16^ Although it seems likely that the parent experience of witnessing immediate neonatal care and resuscitation at birth would be similar when the larger standard resuscitation equipment is used, this merits further research.

This is the first study to explore, in-depth, parents’ experiences of neonatal care being provided beside the mother. Trustworthiness was enhanced by the use of a well-established and appropriate form of analysis, ensuring that participants were given adequate opportunity to refuse participation in the study, the encouragement of a rapport between interviewer and interviewee, frequent debriefing sessions between the team members and a discussion of results with peers who were not part of the research team. Limitations of the study include that participants were primarily white, married or living with their partner, the babies were all alive at the time of interview and most had not required advanced resuscitation at birth, parents were recruited from a single site that had pioneered this type of care, and they were interviewed in hospital before their baby had been discharged. Future research should assess whether the experiences reported here are applicable to parents from different backgrounds, and whether the same experiences are reported for bedside care in other hospitals and using the standard resuscitation equipment. It would be important to include larger numbers of parents who have witnessed advanced resuscitation, as they may be more at risk of negative impact. It is also possible that parents may have been reluctant to criticise staff care or use of the trolley, particularly as staff at this hospital had helped develop the mobile trolley, and most of the interviews were conducted in the hospital. Although this study did not include parents whose baby had died, a related study of clinicians’ experiences suggests bedside care may also be appreciated by parents in these circumstances (CW Yoxall, *et al.* submitted), but it is important that this is explored in future work. It is also important to recognise that parents’ experiences may change over time and they might report later benefits or adverse effects.

Finally, the aim of the study was not to compare neonatal care/resuscitation at birth beside the mother compared with neonatal care/resuscitation at birth away from her, but rather to describe, in detail, parents’ experiences of bedside care at birth. Future research could extend this work by conducting a comparative study of the two types of care.

## Conclusions

Our findings suggest that bedside care is valued by parents as it allows them to see and touch their baby at birth so they are involved in the first moments of their baby's life, and provides reassurance that they know what is happening to their baby and that staff are doing the best they can. Although some parents felt they might not have wanted to watch their baby being resuscitated, parents for whom this happened said they would recommend it to others, even if they found the experience difficult. Further research is needed to assess whether parents’ experiences are similar in other hospitals, and to ensure that witnessing advance resuscitation is not associated with negative effects. Better understanding of parents’ needs if neonatal care is provided at the bedside is required, so that appropriate procedures and support can be developed and evaluated. It is also important to explore how parents’ views change over time, as they have more time to reflect on their experience.
